# Overproduction of *Toxoplasma gondii* cyclophilin-18 regulates host cell migration and enhances parasite dissemination in a CCR5-independent manner

**DOI:** 10.1186/1471-2180-14-76

**Published:** 2014-03-25

**Authors:** Hany M Ibrahim, Maki Nishimura, Sachi Tanaka, Walaa Awadin, Hidefumi Furuoka, Xuenan Xuan, Yoshifumi Nishikawa

**Affiliations:** 1 National Research Center for Protozoan Diseases, Obihiro University of Agriculture and Veterinary Medicine, Inada-cho, Obihiro, Hokkaido 080-8555, Japan; 2 Division of Pathobiological Science, Department of Basic Veterinary Medicine, Obihiro University of Agriculture and Veterinary Medicine, Obihiro 080-8555, Japan; 3 Zoology Department, Faculty of Science, Minufiya University, Shebeen, El Kom, Egypt; 4 Department of Pathology, Faculty of Veterinary Medicine, Mansoura University, Mansoura, Egypt

**Keywords:** *Toxoplasma gondii*, Cyclophilin 18, Cysteine-cysteine chemokine receptor 5, CD11b^+^ cells, Macrophage migration, Host cell recruitment

## Abstract

**Background:**

*Toxoplasma gondii* hijacks host cells to allow it to disseminate throughout a host animal; however, the migratory machinery involved in this process has not been well characterized. We examined the functional role of *T. gondii* cyclophilin 18 (TgCyp18) in host cell recruitment using recombinant parasites transfected with TgCyp18.

**Results:**

High levels of TgCyp18 enhanced IL-12 production in cysteine-cysteine chemokine receptor 5 (CCR5) knockout mice (CCR5^−/−^) that had been infected peritoneally with *T. gondii*. Recruitment of CD11b^+^ cells to the infection site was enhanced in a CCR5-independent manner. *T. gondii* spread to several organs, particularly the liver, in a TgCyp18-dependent and CCR5-independent manner. Additionally, CCL5 levels were upregulated in macrophages treated with recombinant protein TgCyp18 and in the peritoneal fluids of the infected CCR5^−/−^ mice. Furthermore, the chemokines involved in macrophage migration, CCL2 and CXCL10, were upregulated in the livers of CCR5^−/−^ mice infected with recombinant parasites that had been transfected with TgCyp18.

**Conclusion:**

TgCyp18 may play a crucial role in macrophage migration, and in assisting with transport of *T. gondii* via CCR5-independent mechanisms. TgCyp18 may also play a role with CCL5 in the migration of macrophages to the site of infection, and with CCL2 and CXCL10 in the transport of *T. gondii*-infected cells to the liver.

## Background

*Toxoplasma gondii* is an obligate intracellular protozoan parasite that can invade and replicate in the nucleated cells of many animal species, including humans. In several host species, *T. gondii* is associated with congenital infection and abortion [1], and it can also cause encephalitis or systemic infections in immunocompromised individuals, particularly those with AIDS [2]. *T. gondii* can affect pro- and anti-inflammatory host cell signaling in such a way as to maximize parasite multiplication and spread, while maintaining host survival [3]. An aspect of this is the up-regulation of interleukin-12 (IL-12)-dependent production of interferon gamma (IFN-γ), which is critical for host survival during acute toxoplasmosis [4,5]. To perform this essential role in host defense, immune cells must migrate to the site of infection, where they release IFN-γ, which is critical for macrophage and T cell activation [6].

Leukocytes are used by *T. gondii* for transport throughout a host animal [7]. When a host ingests *T. gondii*-containing cysts or oocysts, free parasites are released into the gut lumen. After invading enterocytes, infected cells secrete chemokines such as chemokine (C-C motif) ligand 2 (CCL2), CCL3, CCL4, and chemokine (C-X-C motif) ligand 2 (CXCL2), to recruit leukocytes into the lamina propria extravascular space [8]. The parasites then spread to several distant tissues such as the spleen, lungs and brain [9] and *T. gondii*-infected CD11b^+^ leukocytes actively travel through the lymphatic system and blood vessels [7].

*T. gondii* possesses a unique mechanism for stimulating immune responses and cell migration in the host. Profilin, a *T. gondii* actin binding protein, enhances the production of IL-12 via myeloid differentiation protein-88 (MyD88) and toll-like receptor (TLR) 11 [10]. It has been reported that *T. gondii* heat shock protein 70-induced nitric oxide (NO) release was dependent on TLR2, MyD88 and the IL-1 receptor-associated kinase 4 [11]. This immunomodulatory effect also involves cysteine-cysteine chemokine receptor 5 (CCR5) triggering in dendritic cells (DCs) and macrophages, through the secretion of *T. gondii* cyclophilin (TgCyp18) [12-14]. TgCyp18 appears to induce IL-12 production by interacting directly with CCR5. This effect can be blocked by cyclosporin A [13,15,16], suggesting that this is a unique property of TgCyp18.

Interestingly, TgCyp18 recruits immature mouse DCs *in vitro*; it appears to act as a structural mimic of CCR5-binding ligands, albeit one with no sequence similarity to known host ligands (CCL3, CCL4, CCL5 or CCL8) for this receptor [12,15,16]. In a previous study, we showed that TgCyp18 controlled the migration of macrophages and spleen cells *in vitro* in a dose- and CCR5-dependent manner [14]. However, our *in vitro* studies also showed that cytokine production and macrophage proliferation occurred in a CCR5-independent manner [13,14]. Therefore, elucidation of TgCyp18 functions in regard to *T. gondii* dissemination throughout a host will be important for understanding transport mechanisms in host cells and parasites. This study, therefore, aimed to investigate the role of TgCyp18 in cellular recruitment and parasite dissemination in a CCR5-independent manner through the use of recombinant parasites that had been transfected with TgCyp18.

## Methods

### Ethics statement

This study was performed in strict accordance with the recommendations in the Guide for the Care and Use of Laboratory Animals of the Obihiro University of Agriculture and Veterinary Medicine. The protocol was approved by the Committee on the Ethics of Animal Experiments of the Obihiro University of Agriculture and Veterinary Medicine (Permit number 24–15, 25–59). All surgery was performed under isoflurane anesthesia, and all efforts were made to minimize animal suffering.

### Parasite and cell cultures

The RH strain of *T. gondii* and its recombinant derivatives were maintained in Vero (African green monkey kidney epithelial) cells cultured in Eagle’s minimum essential medium (EMEM; Sigma, St Louis, MO) supplemented with 8% heat-inactivated fetal bovine serum (FBS, Nichirei Biosciences, Tokyo, Japan). For tachyzoite purification, parasites and host-cell debris were washed in cold phosphate-buffered saline (PBS), and the final pellet was resuspended in cold PBS, then passed through a 27-gauge needle and a 5.0-μm-pore filter (Millipore, Bedford, MA).

### Animals

Female C57BL/6 J mice were obtained from CLEA Japan (Tokyo, Japan). CCR5 knockout mice (CCR5^−/−^, B6.129P2-*Ccr5*^*tm1Kuz*^/J, Stock No. 005427) were purchased from the Jackson laboratory (Bar Harbor, ME). Animals were housed under specific pathogen-free conditions in the animal facility at the National Research Center for Protozoan Diseases (Obihiro University of Agriculture and Veterinary Medicine, Obihiro, Japan). Animals used in this study were treated and used according to the Guiding Principles for the Care and Use of Research Animals published by the Obihiro University of Agriculture and Veterinary Medicine.

### Transfer vector construction

cDNA synthesized from RNA isolated with TRI reagent (Sigma) using a SuperScript™ First-strand Synthesis System for RT-PCR (Invitrogen, Carlsbad, CA) was used as a template to amplify the coding region of the full-length TgCyp18 gene (GenBank accession number U04633.1). The primers used to amplify the TgCyp18 gene contained the *Nco*I recognition sequence (boldface) in the forward primer (5′-AG**C CAT GG**A TGA AGC TCG TGC TGT TTT TC-3′) and a *Nhe*I site (boldface) in the reverse primer (5′-GT**G CTA GC**C TCC AAC AAA CCA ATG TCC GT-3′). Amplicons were digested with *Nco*I and *Nhe*I and then ligated into pCR4-TOPO (Invitrogen) to yield pCR4-TOPO-TgCyp18. The nucleotide sequences of recombinant plasmids were analyzed with an ABI 3100 DNA sequencer (Applied Biosystems, Foster City, CA). The pCR4-TOPO-TgCyp18 construct was digested with *Nco*I and *Nhe*I and the resulting product ligated into pHXNTPHA (kindly provided by K.A. Joiner, Yale University), resulting in the plasmid, pHXNTP-TgCyp18HA. Coding sequences corresponding to the full-length TgCyp18 fused to hemagglutinin (HA) were obtained from pHXNTP-TgCyp18HA by *Nco*I and *Bgl*II digestion. Liberated fragments were treated with the Klenow fragment of DNA polymerase I and then inserted into the *Eco*RV site of pDMG [17]. The pDMG-TgCyp18HA vector contained expression cassettes for the green fluorescent protein (GFP), dihydrofolate (DHFR)-thymidylate synthase (TS) and TgCyp18-HA.

### Transfection and selection of *T. gondii*

Electroporation of tachyzoites was performed as previously described [18]. Briefly, purified *T. gondii* RH tachyzoites were resuspended (10^7^ cells/ml) in cytomix buffer (120 mM KCl, 0.15 mM CaCl_2_, 10 mM K_2_HPO_4_-KH_2_PO_4_, 2 mM EDTA, 5 mM MgCl_2_, 25 mM HEPES, pH 7.6) supplemented with 2 mM adenosine triphosphate (ATP) and 5 mM glutathione. Cells were electroporated (2.0 kV at 50 W) using a Gene Pulser II (BioRad Laboratories, Tokyo Japan). After transfection, tachyzoites were allowed to infect Vero cells for 18 h in drug-free culture medium to permit phenotypic expression of the DHFR-TS and GFP genes as selectable markers, after which pyrimethamine was added at a final concentration of 1 μM. Polyclonal transfected pyrimethamine-resistant tachyzoite cultures were subjected to plaque purification. Cultures were passaged at least four times in the same medium containing 1% agarose and a single plaque was obtained. Positive clones were identified by indirect fluorescent antibody tests (IFATs) using an anti-HA.11 mouse monoclonal antibody (mAb; Covance, Emeryville, CA). The resultant recombinant *T. gondii* clones, pDMG-TgCyp18HA and pDMG, are hereafter designated RH-OE and RH-GFP, respectively. The TgCyp18 expression levels among three independent clones from each transfectant were examined by western blotting and TgCyp18 secretion assays, and a representative clone was selected for further study.

### Western blot analysis

Tachyzoites (1 × 10^6^) of wild type parasites (RH-WT), RH-OE or RH-GFP were harvested, washed and suspended in 10 μl of PBS, sonicated, and then mixed with 10 μl of 2 × sodium dodecyl sulfate (SDS) gel-loading buffer [62.5 mM Tris–HCl pH 6.8, 2% (w/v) SDS, 140 mM 2-mercaptoethanol, 10% (w/v) glycerol and 0.02% (w/v) bromophenol blue] under reducing conditions. Samples were heated at 95°C for 5 min and separated on a 15% polyacrylamide gel. After SDS polyacrylamide gel electrophoresis the protein bands in the gel were transferred to a nitrocellulose membrane (Whatman GmbH, Dassel, Germany). After washing twice with PBS containing 0.05% (v/v) Tween 20 (PBS-T), membranes were blocked with PBS containing 3% (w/v) skimmed milk (PBS-SM) for 12 h at 4°C. After two further washes, the membranes were incubated with an anti-TgCyp18 rabbit antibody at 1:500 [13], an anti-TgSAG1 mAb (1:1000; Advanced Immunochemical Inc., Long Beach, CA) or an anti-HA.11 mAb (1:1000; Covance) for 1 h at room temperature. After washing three times, the membranes were incubated with horseradish peroxidase (HRP)-conjugated goat anti-mouse immunoglobulin G (1:1000; Amersham Pharmacia Biotech, Piscataway, NJ) diluted in PBS-SM, for 1 h at 37°C. After washing three times, the proteins were visualized on X-ray film using ECL™ western blotting detection reagents (GE Healthcare UK Ltd., Buckinghamshire, UK) according to the manufacturer’s recommendations.

### Parasite infections in mice

Parasites purified from *in vitro* cultures were washed in sterile PBS and tachyzoites (5 × 10^2^ – 1 × 10^3^) were inoculated intraperitoneally into mice. Three or five days after the infection, cells were collected from the peritoneal cavity of naïve or parasite-infected mice by peritoneal washing with 5 ml of cold PBS. After harvesting, the cells were centrifuged at 800 × *g* for 10 min and suspended in cold PBS. These cells were then subjected to flow cytometry. Supernatants were used to measure TgCyp18, IL-12, CCL2, CCL5 and CXCL10 production. To determine the parasite burden and chemokine expression levels in the mice, tissues including the brain, liver, lungs and spleen from *T. gondii* infected and uninfected animals were collected at 0, 3 and 5 days post-infection (dpi).

### Sandwich enzyme-linked immunosorbent assay (ELISA) detection of TgCyp18

The presence of TgCyp18 in mouse ascites fluid and TgCyp18 secreted by extracellular parasites in infected mice was determined by a sandwich ELISA as described previously [14]. To detect TgCyp18 from extracellular tachyzoites, purified *T. gondii* tachyzoites (3 × 10^7^) were incubated in 1.5 ml of GIT medium (Nihon Pharmaceutical Co., Ltd, Tokyo, Japan) at 37°C. Before transferring parasite suspensions from ice to 37°C for a secretion assay, 250 μl of the parasite suspension was removed and processed as the time zero reading. The remainder of the parasite suspension was incubated at 37°C in a water bath. After 15, 30, 60, and 120 min, 250 μl of parasite suspension was removed. The culture supernatants were centrifuged (760 × *g* for 10 min at 4°C, then 7000 × *g* for 10 min at 4°C) together with the ascites fluid from the *in vivo* experiment, and then subjected to sandwich ELISA.

Microtiter plates were coated with 1 μg of rabbit anti-rTgCyp18 polyclonal IgG [13] diluted in 0.05 M carbonate buffer (pH 9.6), which was used as the capture antibody at 4°C overnight. Blocking was performed with a blocking solution (PBS-SM, pH 7.2) at 37°C for 2 h. Microtiter plates were incubated at 37°C for 30 min with each supernatant in triplicate. After washing six times with PBS-T, anti-TgCyp18 mouse serum (1:100) was added to each well as the detection antibody. After a further six washes, the plates were incubated with HRP-conjugated goat anti-mouse IgG (1:2500; Amersham Pharmacia Biotech). Binding was visualized with substrate solution [0.3 mg/ml 2,2'-azino-bis-(3-ethylbenz-thiazoline-6-sulfonic acid), 0.1 M citric acid, 0.2 M sodium phosphate, 0.003% H_2_O_2_]. Absorbance at 415 nm was measured using a MTP-500 microplate reader (Corona Electric, Tokyo, Japan). The TgCyp18 concentration in each sample was calculated by standardization against the recombinant TgCyp18 protein [13].

### Cytokine ELISA

Ascetic fluid was collected for measurement of total IL-12, CCL2, CCL5 and CXCL10 levels using ELISA kits (IL-12: Pierce Biotechnology Inc., Rockford, IL; CCL2, CCL5 and CXCL10: R&D Systems, Minneapolis, MN) according to the manufacturer’s recommendations.

### Flow cytometry

Anti-mouse CD11b mAb, anti-mouse CCR5 mAb, anti-mouse CD3e (CD3ϵ chain) mAb, and hamster anti-mouse CD11c (HL3) mAb were purchased from BD Biosciences (San Jose, CA) and labeled with phycoerythrin (PE). After washing with cold PBS, peritoneal cells were suspended in cold PBS containing 0.5% bovine serum albumin, treated with Fc Block™ (BD Biosciences, San Jose, CA, USA) and subsequently incubated with PE-labeled anti-mouse antibodies for 30 min at 4°C followed by a final washing step with cold PBS. *T. gondii*-infected cells were GFP^+^. Labeled cells (1 × 10^4^) were examined using an EPICS® XL flow cytometer (Beckman Coulter, Hialeah, FL). The absolute number of each marker indicated below was calculated as follows: the absolute cell number = the total host cell number × (the percentage of marker^+^ cells/100) × (the percentage of gated cells observed by flow cytometry/100). Infected cells in peritoneal fluids were detected by double signals, comprising CCR5^+^, CD11b^+^, CD11c^+^ or CD3^+^ cell markers labeled with PE using anti-CCR5, anti-CD11b, anti-CD11c and anti-CD3 mAbs, and GFP signaling of the parasites.

### DNA isolation and quantitative PCR (qPCR) detection of *T. gondii*

Tissues (brain, liver, lungs and spleen) and peritoneal fluids from *T. gondii*-infected animals were collected at 0, 3 and 5 dpi. DNA was extracted from tissues by resuspending the samples in extraction buffer (0.1 M Tris–HCl pH 9.0, 1% SDS, 0.1 M NaCl, 1 mM EDTA, 1 mg/ml proteinase K) followed by incubation at 55°C. DNA was purified by phenol-chloroform extraction and ethanol precipitation. Amplification of parasite DNA was performed using primers specific for the *T. gondii B1* gene (5'-AAC GGG CGA GTA GCA CCT GAG GAG A-3' and 5'-TGG GTC TAC GTC GAT GGC ATG ACA AC-3'), which is present in all known strains of this species of parasite [19]. The PCR mixture (25 μl) contained 1 × SYBR Green PCR Buffer, 2 mM MgCl_2_, 200 μM each dNTP, 400 μM dUTP, 0.625 U of AmpliTaq Gold DNA polymerase, and 0.25 U of AmpErase uracil-*N*-glycosylase (UNG) (AB Applied Biosystems, Carlsbad, CA), 0.5 μ moles of each primer and 50 ng of genomic DNA. Amplification was performed by a standard protocol recommended by the manufacturer (2 min at 50°C, 10 min at 95°C, then 40 cycles of 95°C for 15 s and 60°C for 1 min). Amplification, data acquisition, and data analysis were carried out in an ABI 7900HT Prism Sequence Detector (AB Applied Biosystems), and cycle threshold values (Ct) were exported to Microsoft Excel for analysis. Parasite loads were estimated by comparison with internal controls, with the level of the internal control calculated per parasite [20]. Briefly, numbers of parasites were calculated by interpolation on a standard curve, with Ct values plotted against a known concentration of parasites. After amplification, PCR product melting curves were acquired via a stepwise temperature increase from 60°C to 95°C. Data analyses were conducted with Dissociation Curves version 1.0 f (AB Applied Biosystems).

### Peritoneal macrophage cultures

Mouse peritoneal macrophages were collected from mice four days after their intraperitoneal injections with 1 ml of 4.05% brewer modified BBL™ thioglycolate medium (Becton Dickinson, Sparks, MD). Collected cells were washed with 5 ml of cold PBS, then centrifuged at 800 × *g* for 10 min and suspended in RPMI 1640 medium (Sigma) containing 10% FBS. The macrophage suspension was then added to 24-well tissue culture microplates (1 × 10^6^ cells/well). Suspensions were incubated at 37°C for 3 h, washed thoroughly to remove non-adherent cells, and incubated further at 37°C. Macrophages were treated with purified TgCyp18 recombinant protein [13] at 37°C for 20 h. Cells were then harvested for qPCR analysis to determine their chemokine expression levels.

### qPCR analysis of chemokine expression

Total RNA was extracted from cells or homogenized tissues using Tri reagent (Sigma). Reverse transcription of RNA was performed using Superscript II Reverse Transcriptase (Gibco BRL) in a final volume of 25 μl. qPCR was carried out as described above. The relative amounts of all mRNAs were calculated using the comparative Ct method (Perkin-Elmer). Glyceraldehyde-3-phosphate dehydrogenase (GAPDH) mRNA was used as a control. Specific primer sequences for mouse CCL2 (5'-GGC TCA GCC AGA TGC AGT TAA-3' and 5'-CCT ACT CAT TGG GAT CAT CTT GCT-3'), mouse CCL3 (5'-CCA GCC AGG TGT CAT TTT TCC T-3' and 5'-TCC AAG ACT CTC AGG CAT TCA GT-3'), mouse CCL4 (5'-CTC CAA GCC AGC TGT GGT ATT C-3' and 5'-CTC CAA GTC ACT CAT GTA ACT CAG TGA-3'), mouse CCL5 (5'-CCA ATC TTG CAG TCG TGT TTG T-3' and 5'-CAT CTC CAA ATA GTT GAT GTA TTC TTG AAC-3'), mouse CCL6 (5'-TGC CAC ACA GAT CCC ATG TAA-3' and 5'-TGA TGC CCG GCT TGA TG-3'), mouse CCL12 (5'-GAG AAT CAC AAG CAG CCA GTG T-3' and 5'-GCA CAG ATC TCC TTA TCC AGT ATG G-3'), mouse CXCL10 (5'-GAC GGT CCG CTG CAA CTG-3' and 5'-CTT CCC TAT GGC CCT CAT TCT-3'), mouse CX3CL1 (5'-CCG AGG CAC AGG ATG CA-3' and 5'-TGT CAG CCG CCT CAA AAC TT-3'), and mouse GAPDH (5'-TGT GTC CGT CGT GGA TCT GA-3' and 5'-CCT GCT TCA CCA CCT TCT TGA T-3') were designed using Primer Express (Applied Biosystems).

### Statistical analysis

Data are expressed as the mean ± the standard deviation, or as scatter diagrams. Various assay conditions were evaluated using a Student’s *t*-test or an analysis of variance (ANOVA) test followed by Tukey’s multiple comparison. *P < 0.05, **P < 0.01, ***P < 0.001.

## Results

### Characterization of recombinant *T. gondii*

**Figure 1:**
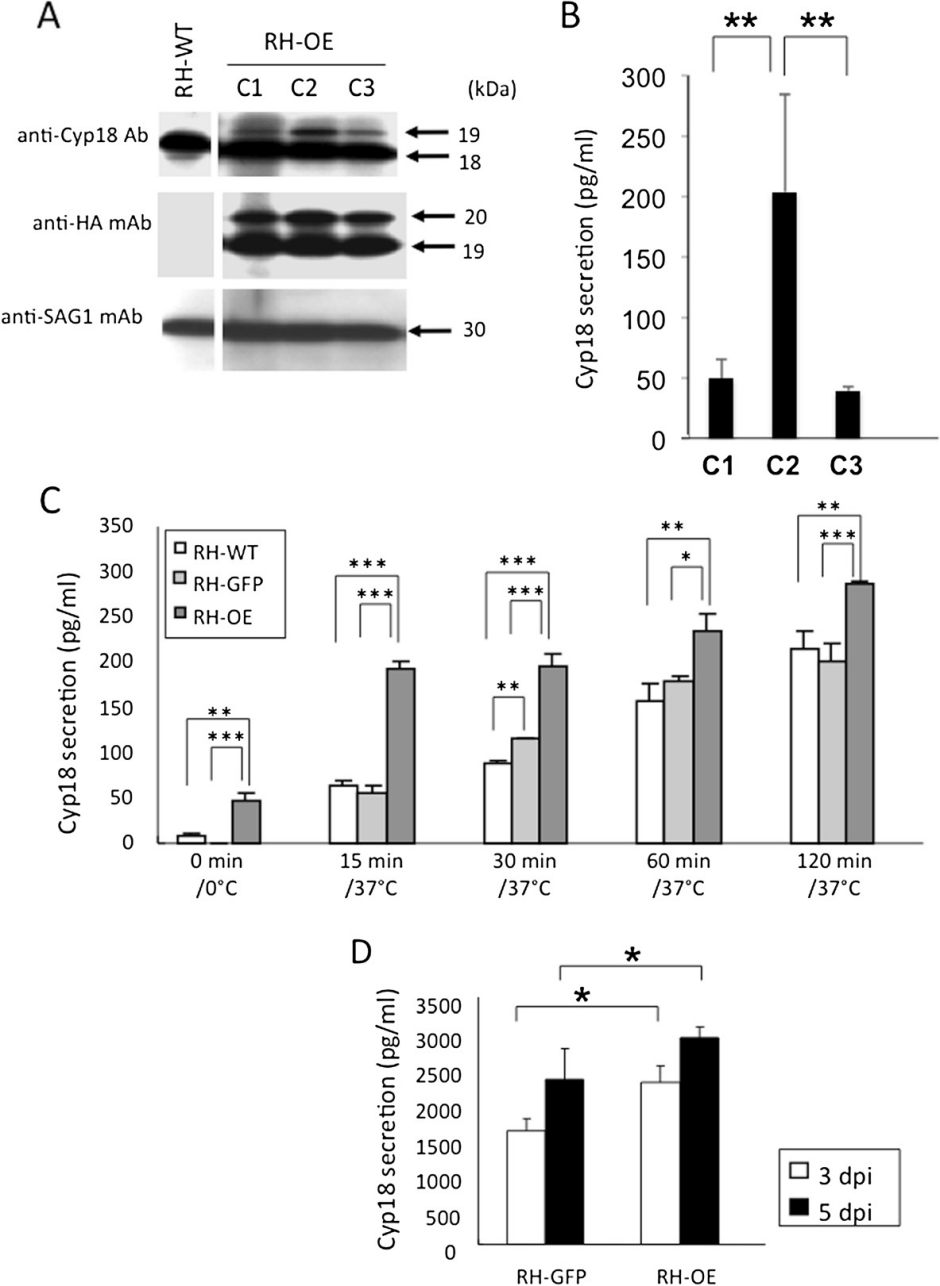
**Characterization of recombinant parasites. (A)** Western blot analysis of *T. gondii* tachyzoites of RH-WT and RH-OE clones (C1, C2 and C3). **(B)** Secretion of TgCyp18 from extracellular parasites of RH-OE clones at 30 min incubation. Each value represents the mean ± the standard deviation of triplicate samples. **(C)** Secretion of TgCyp18 from RH-WT, RH-GFP and RH-OE (clone C2) extracellular parasites. Each value represents the mean ± the standard deviation of triplicate samples. **(D)** TgCyp18 secretion in the ascetic fluid of infected mice at 3 and 5 days post-infection (dpi). Tachyzoites were inoculated intraperitoneally into wild type mice. Each value represents the mean ± the standard deviation of four replicate samples. Results are representative of two repeated experiments with similar results. RH-WT: wild-type parasites; RH-GFP: parasites transfected with GFP; RH-OE: parasites transfected with TgCyp18HA and GFP.

Recombinant parasites expressing TgCyp18 fused to HA were established. Three independent clones expressing TgCyp18-HA were isolated from transfected polyclonal cultures. The reactivity of the recombinant parasites to an anti-HA.11 mAb and GFP were confirmed by IFATs. IFAT analyses showed that TgCyp18-HA and GFP expression was detected within the parasite cytosol of the intracellular parasites (data not shown). In addition, HA expression was not observed in *T. gondii* expressing GFP (RH-GFP) or in wild type parasites (data not shown). Western blot analysis was performed to confirm expression of endogenous TgCyp18 and transfected TgCyp18-HA (Figure 1A). An anti-SAG1 antibody was used as an internal control to confirm that each lane contained an equal amount of parasite lysate. Western blotting with an anti TgCyp18 antibody indicated that the three pDMG-TgCyp18HA clones (used to produce RH-OE parasites) each expressed an additional band of a slightly larger size (19 kDa) than that of the endogenous protein (18 kDa), as shown in RH-WT (Figure 1A) and RH-GFP (data not shown). Expression of TgCyp18-HA from RH-OE was confirmed using the anti-HA.11 mAb. Reactivity against anti-HA.11 mAb was not seen in RH-WT (Figure 1A) and RH-GFP parasites (data not shown). The 19 kDa band was seen in the three RH-OE clones. The band at 19 kDa was consistent with that observed on the anti-TgCyp18 western blot. The band at 20 kDa, seen in the three RH-OE clones, might be premature TgCyp18-HA. Furthermore, there was no significant difference in the growth of RH-GFP clones, or the three RH-OE clones in Vero cells (data not shown). In a TgCyp18 secretion assay, the C2 clone produced more TgCyp18 protein than the other clones (Figure 1B). Thus, the RH-OE C2 clone was selected for further studies.

### Detection of TgCyp18 in extracellular parasites and infected mice

Extracellular RH-OE parasites spontaneously secreted higher levels of TgCyp18 into the medium compared with RH-WT or RH-GFP (Figure 1C). Time-dependent secretion of TgCyp18 by the extracellular parasites was observed. In addition, statistically significant higher levels of TgCyp18 were detected in the ascetic fluid from RH-OE-infected mice at 3 and 5 dpi compared with that of the RH-GFP-infected animals (Figure 1D).

### Effects of TgCyp18 induction on IL-12 production *in vivo*

**Figure 2:**
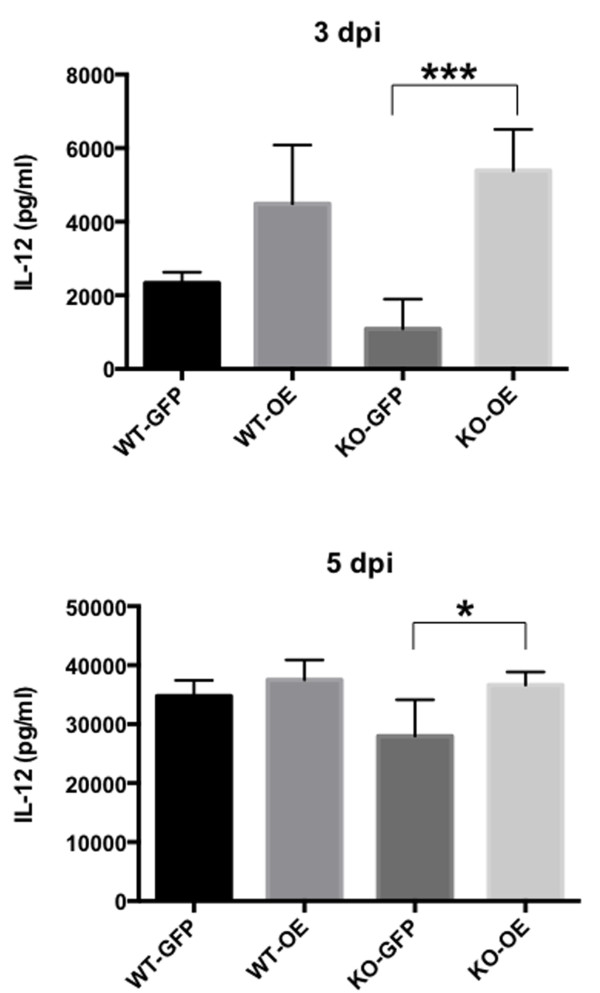
**IL-12 production in the ascites fluid of infected mice.** Wild type (WT) and CCR5^−/−^ (KO) mice were infected intraperitoneally with *T. gondii* tachyzoites. IL-12 production in the ascites fluid was measured at 3 and 5 days post-infection (dpi). Each value represents the mean ± the standard deviation of four replicate samples. RH-GFP (GFP): parasites transfected with GFP; RH-OE (OE): parasites transfected with TgCyp18HA and GFP. Results are representative of two repeated experiments with similar results.

Upon *in vitro* infection with RH-OE parasites, IL-12 production was not significantly different in the infected peritoneal macrophages than those infected with RH-GFP parasites (data not shown). To compare cytokine production between the WT and CCR5^−/−^ mice following *T. gondii* infection, ascetic fluid was collected from RH-GFP- and RH-OE-infected animals (Figure 2). Significant increases in IL-12 production were apparent in the CCR5^−/−^ mice infected with RH-OE at 3 and 5 dpi compared with infections with RH-GFP. However, there was no significant difference in IL-12 production levels between WT and CCR5^−/−^ mice infected with the same parasite strain.

### Effects of TgCyp18 on immune cell recruitment

**Figure 3:**
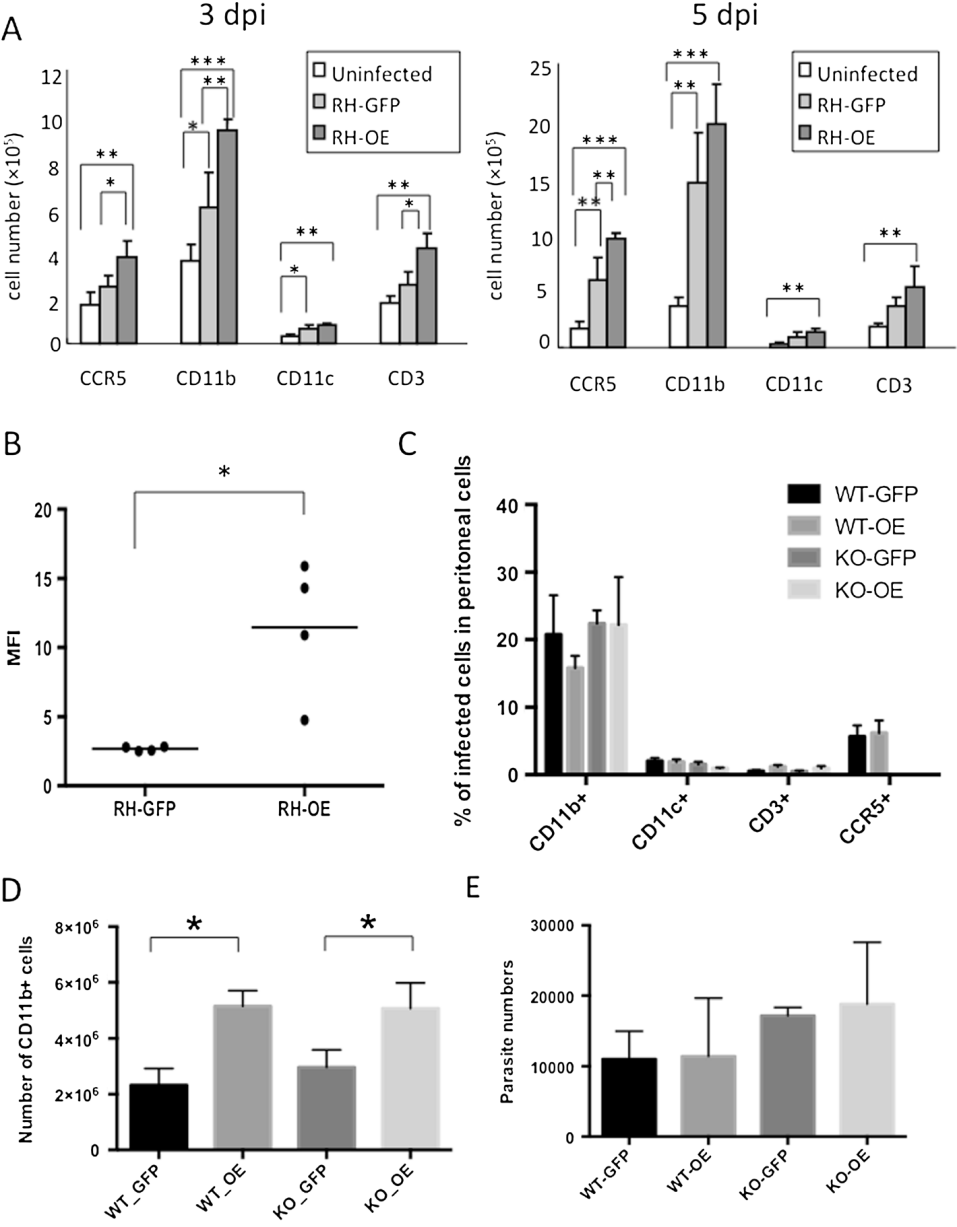
**Immune cell recruitment and parasite infections. (A)** Wild type (WT) mice were infected intraperitoneally with *T. gondii* tachyzoites. Peritoneal cells were harvested from uninfected or parasite-infected mice at 3 and 5 days post-infection (dpi). Cells were then subjected to flow cytometry to determine the absolute number of cells expressing CCR5, CD11b, CD11c, or CD3. Each value represents the mean ± the standard deviation of four replicate samples. **(B)** CCR5 expression levels in peritoneal cells at 3 dpi. WT mice were infected intraperitoneally with *T. gondii* tachyzoites. CCR5^+^ and GFP^+^ host cells were detected using flow cytometry and the mean fluorescence intensity (MFI) of CCR5 expression was determined. Infection rates for RH-GFP and RH-OE were 50.9 ± 5.4% and 50.4 ± 4.1%, respectively. Bars represent the average for each experimental group (*n* = 4). **(C)** Peritoneal cell infection rates. WT and CCR5^−/−^ (KO) mice were infected intraperitoneally with *T. gondii* tachyzoites. At 5 dpi, peritoneal cells were subjected to flow cytometry to determine the number of GFP^+^ host cells. Each value represents the mean ± standard deviation of four replicate samples. **(D)** WT and KO mice were infected intraperitoneally with *T. gondii* tachyzoites. At 3 dpi, peritoneal cells were collected and the number of CD11b^+^ cells was measured. Each value represents the mean ± the standard deviation of four replicate samples. **(E)** Real-time PCR quantification of parasites in the peritoneal cells of WT and KO mice at 5 dpi. Each value denotes the number of parasites in 50 ng of DNA and represents the mean ± the standard deviation of four replicate samples. RH-GFP (GFP): parasites transfected with GFP alone; RH-OE (OE): parasites transfected with TgCyp18HA and GFP. The results are representative of two repeated experiments with similar results.

Absolute numbers of CD11b^+^ (monocyte/macrophage), CD11c^+^ (DC), CD3^+^ (T cells) and CCR5^+^ cells recruited to the site of infection were measured (Figure 3A). At both 3 and 5 dpi, RH-GFP infection enhanced the migration of CD11b^+^ cells, while CCR5^+^, CD11b^+^, CD11c^+^ and CD3^+^ cell migration were all enhanced by RH-OE infection. At 3 dpi, CCR5^+^, CD11b^+^ and CD3^+^ cell migration was enhanced in WT mice infected with RH-OE compared with RH-GFP. At 5 dpi, the absolute number of CCR5^+^ cells was significantly different in WT mice infected with RH-OE than in uninfected and RH-GPF-infected mice. A comparison of infection rates for RH-GFP and RH-OE in CCR5^+^ cells showed there was no significant difference between the two strains at 3 dpi (RH-GFP, 50.9 ± 5.4%; RH-OE, 50.4 ± 4.1%). CCR5 expression levels increased in the RH-OE-infected CCR5^+^ cells from mice at 3 dpi (Figure 3B). Further analysis of host cell recruitment was conducted by analyzing the peritoneal cells of WT and CCR5^−/−^ mice infected with RH-GFP or RH-OE at 5 dpi (Figure 3C). *T. gondii* showed CD11b^+^ cell tropism, with no significant difference in the rates of infection (Figure 3C), or the absolute numbers of RH-OE and RH-GFP parasites in these cells (Additional file 1: Figure S1). At 3 dpi, CD11b^+^ cell migration was enhanced in WT and CCR5^−/−^ mice infected with RH-OE compared with those groups infected with RH-GFP (Figure 3D). However the differences were not statistically significant between WT and CCR5^−/−^ mice infected with same parasite strain (Figure 3D). In addition, no significant differences in the numbers of parasites in the peritoneal cavity of the different groups of infected mice at 5 dpi were found (Figure 3E). This chemotactic result was correlated with high levels of TgCyp18 production caused by RH-OE infection.

### Effects of TgCyp18 on parasite trafficking properties

**Figure 4:**
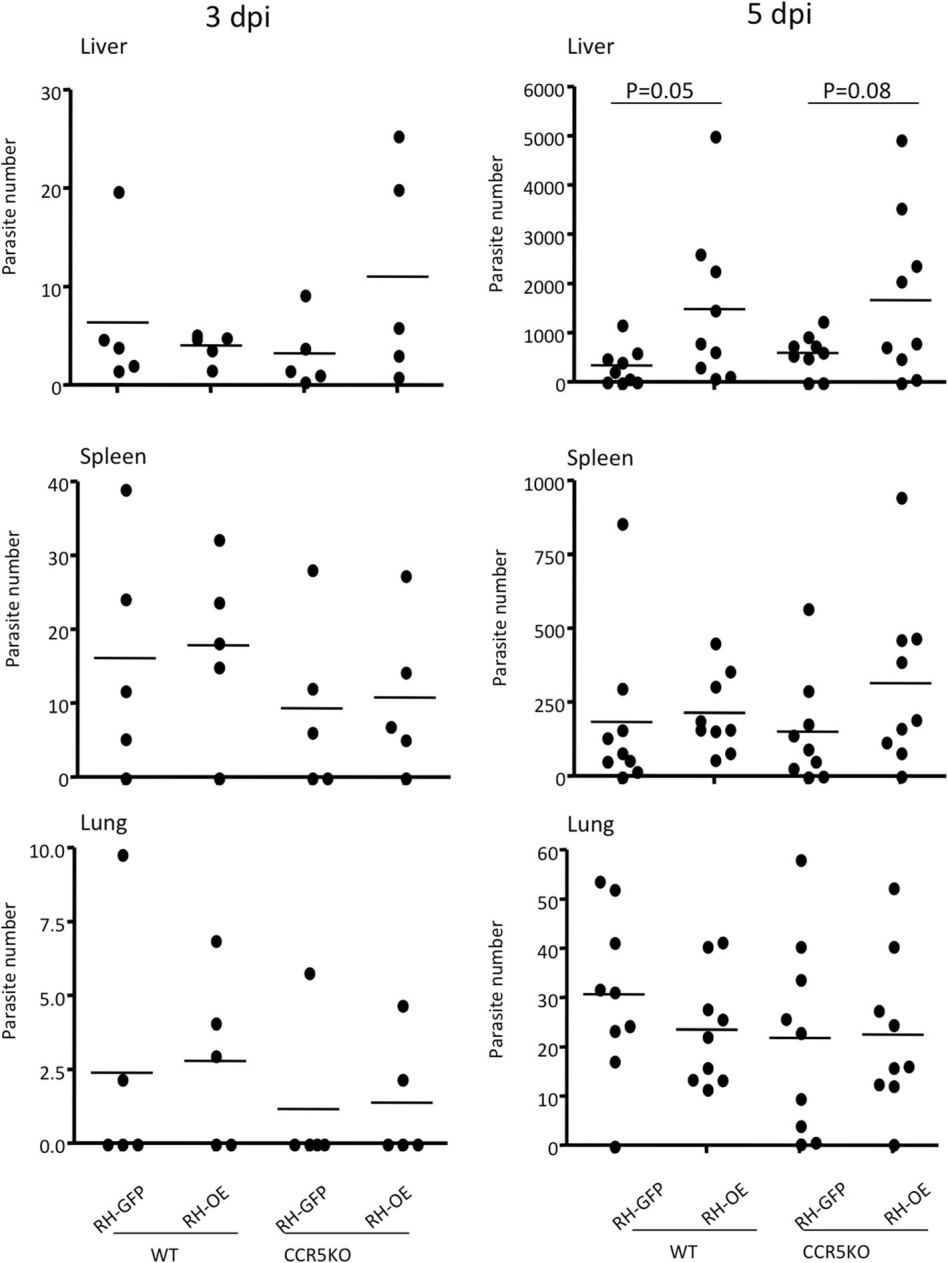
**Parasite load in liver, spleen, and lung tissues of infected mice.** Wild type (WT) and CCR5^−/−^ (CCR5 KO) mice were infected intraperitoneally with *T. gondii* tachyzoites. At 3 and 5 dpi, liver, spleen and lungs were collected and the parasite numbers in 50 ng of DNA determined by quantitative PCR. Bars represent the average for each experimental group (3 dpi, *n* = 5; 5 dpi, *n* = 9). RH-GFP (GFP): parasites transfected with GFP alone; RH-OE (OE): parasites transfected with TgCyp18HA and GFP.

To further elucidate the role of TgCyp18 in trafficking parasite-infected leukocytes, the brains, livers, lungs and spleens from infected animals were collected at 3 and 5 dpi, and the parasite numbers were determined (Figure 4). Parasites were detected at 3 and 5 dpi in the livers, spleens and lungs of mice infected with RH-GFP and RH-OE. Parasites were not detected in brain tissue at 3 and 5 dpi (data not shown). WT and CCR5^−/−^ mice infected with RH-OE had increased parasite loads in the liver compared with the RH-GFP-infected mice. There was no significant difference in parasite load between WT and CCR5^−/−^ mice infected with the same parasite strain.

### Effects of TgCyp18 on expression of the CCR5 ligands and chemokines involved in macrophage migration *in vitro* and *in vivo*

**Figure 5:**
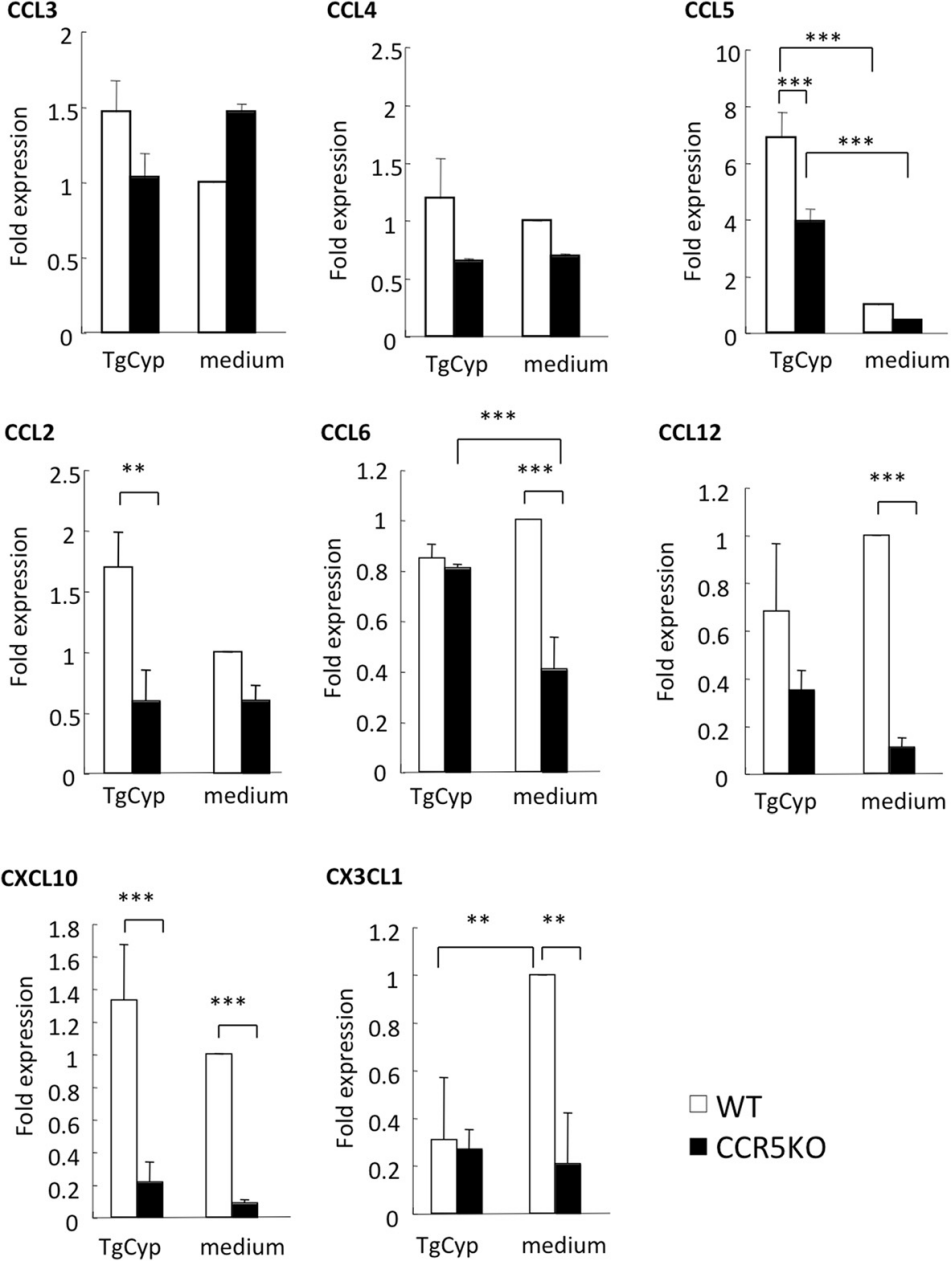
**Chemokine ligand expression.** To analyze expression of CCR5 ligands (CCL3, CCL4 and CCL5), CCL2, CCL6, CCL12, CXCL10, and CX3CL1 by real-time PCR, peritoneal macrophages were treated with recombinant TgCyp18 (TgCyp) or culture medium alone for 20 h. Each value represents the mean ± the standard deviation of triplicate samples.

To investigate the role of TgCyp18 on the expression of CCR5 ligands (CCL3, CCL4 and CCL5), peritoneal macrophages were treated with recombinant TgCyp18 protein *in vitro* (Figure 5). CCL3 and CCL4 expression was not affected by TgCyp18 treatment. However, CCL5 expression was enhanced by TgCyp18, partially in a CCR5-dependent manner. Additionally, we investigated the effects of the TgCyp18 recombinant protein on expression of the chemokines involved in macrophage migration to confirm chemokine expression occurred in a CCR5-independent manner (Figure 5). CCL2 expression was enhanced 2-fold in a CCR5-dependent manner. In the absence of TgCyp18, the expression levels of CCL6, CCL12, CXCL10 and CX3CL1 in CCR5^−/−^ macrophages were significantly lower than those in WT macrophages. CX3CL1 expression was down-regulated by TgCyp18 in a CCR5-dependent manner. CCL6 expression in CCR5^−/−^ macrophages was significantly increased by TgCyp18.

**Figure 6:**
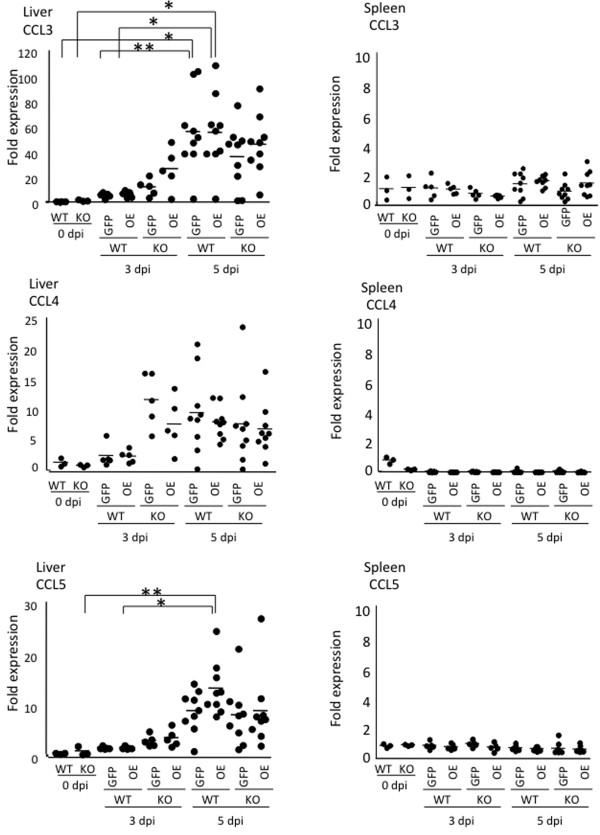
**Expression of CCR5 ligands in liver and spleen tissues from infected mice.** Wild type (WT) and CCR5^−/−^ (KO) mice were infected intraperitoneally with *T. gondii* tachyzoites. Liver and spleen tissues were collected and their total RNA content was isolated at 0, 3 and 5 days post-infection (dpi). Expression of the target mRNA was determined and compared to expression levels at 0 dpi using quantitative PCR. Bars represent the average for each experimental group (0 dpi, *n* = 3; 3 dpi, *n* = 5; 5 dpi, *n* = 9). RH-GFP (GFP): parasites transfected with GFP alone; RH-OE (OE): parasites transfected with TgCyp18HA and GFP.

**Figure 7:**
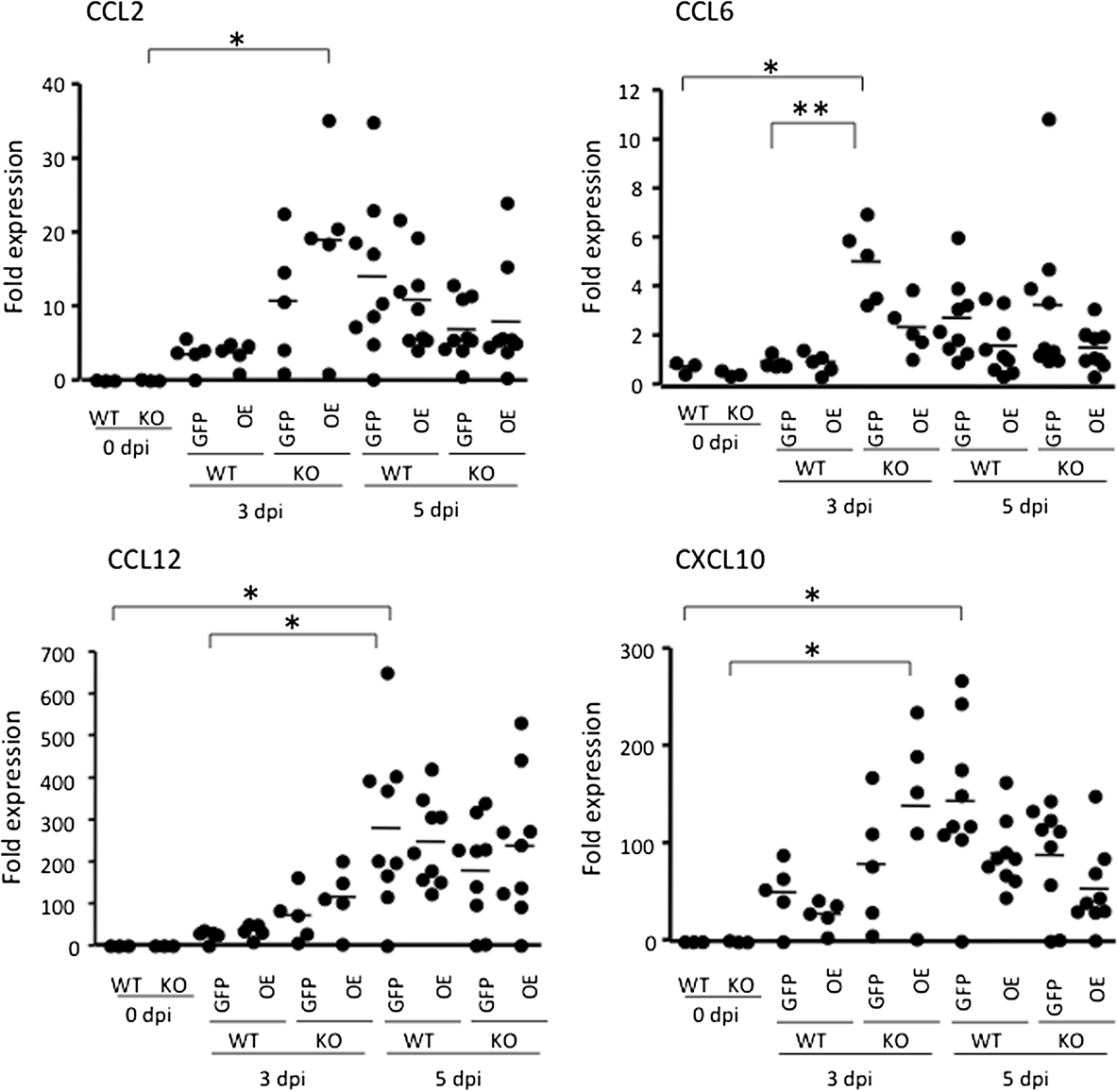
**Expression of the chemokines involved in macrophage migration in the livers of infected mice.** Wild type (WT) and CCR5^−/−^ (KO) mice were infected intraperitoneally with *T. gondii* tachyzoites. Fold expression levels for CCL2, CCL6, CCL12 and CXCL10 mRNA was determined by quantitative PCR. Bars represent the average for each experimental group (0 dpi, *n* = 3; 3 dpi, *n* = 5; 5 dpi, *n* = 9). RH-GFP (GFP): parasites transfected with GFP alone; RH-OE (OE): parasites transfected with TgCyp18HA and GFP.

Next, the spleens and livers of mice infected with RH-GFP and RH-OE were examined *in vivo* (Figure 6). *T. gondii* infection up-regulated expression of CCR5 ligands in the liver, but had no obvious effect on the spleen. In the liver, significantly increased CCL3 expression in WT mice infected with RH-GFP and RH-OE occurred at 5 dpi, while significantly increased CCL5 expression in WT mice infected with RH-OE occurred at 5 dpi, suggesting that CCL5 expression took place in a TgCyp18-dependent manner. As shown in Figure 7, comparisons of CCL2, CCL6, CCL12 and CXCL10 expression *in vivo* indicated that higher CCL2 and CXCL10 expression occurred in the livers of CCR5^−/−^ mice infected with RH-OE at 3 dpi compared with uninfected CCR5^−/−^ mice; this suggests that the TgCyp18-mediated CCL2 and CXCL10 expression occurred in a CCR5-independent way. Moreover, higher levels of CCL6 in the CCR5^−/−^ mice infected RH-GFP at 3 dpi and CCL12 in the WT mice infected with RH-GFP at 5 dpi were detected, compared with the uninfected mice.

**Figure 8:**
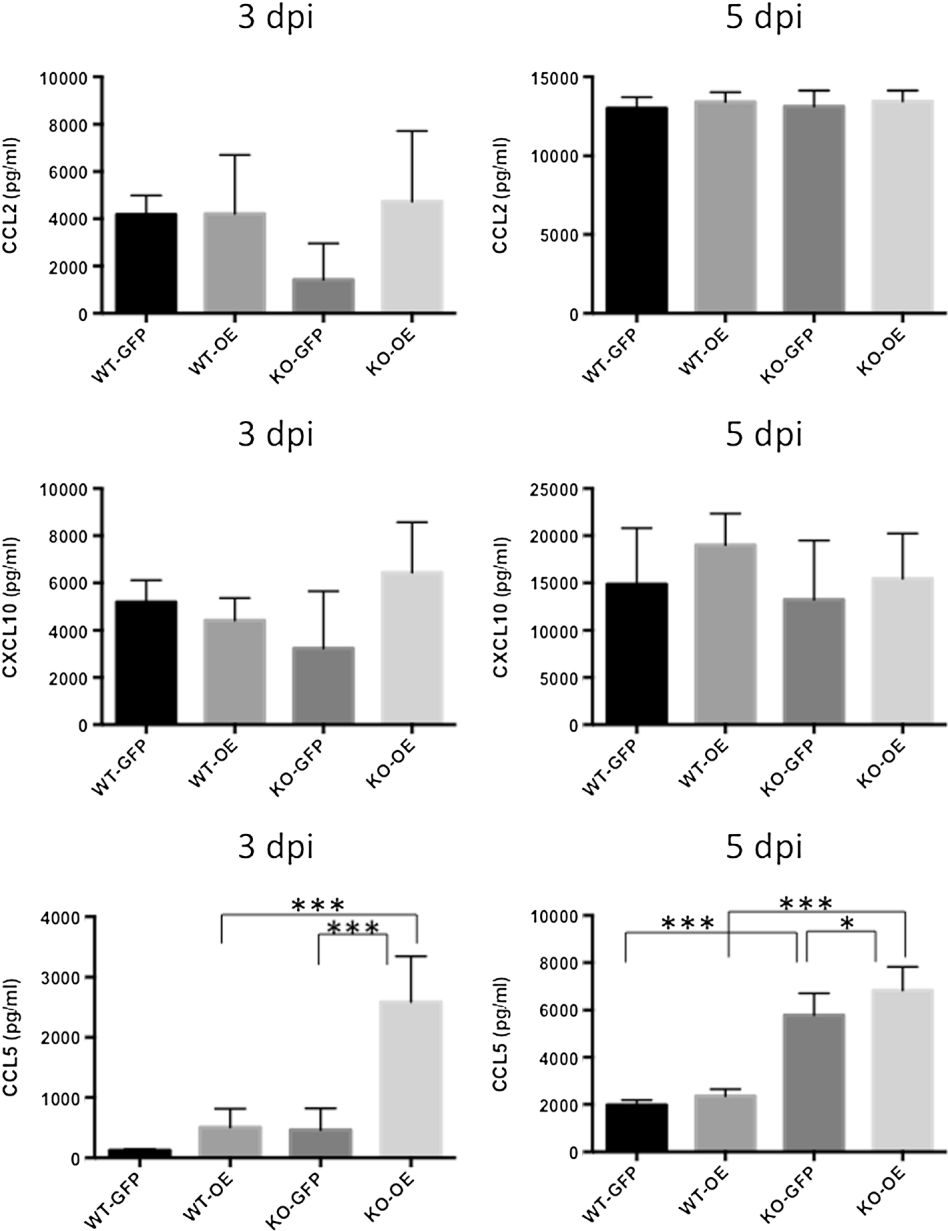
**CCL2, CCL5 and CXCL10 production in the ascites fluid of infected mice.** Wild type (WT) and CCR5^−/−^ (KO) mice were infected intraperitoneally with *T. gondii* tachyzoites. CCL2, CCL5 and CXCL10 production in the ascites fluid was measured at 3 and 5 days post-infection (dpi). Each value represents the mean ± the standard deviation of replicate samples (3 dpi, *n* = 5; 5 dpi, *n* = 9). RH-GFP (GFP): parasites transfected with GFP alone; RH-OE (OE): parasites transfected with TgCyp18HA and GFP.

In view of the results of our *in vitro* and *in vivo* studies, we examined CCL2, CXCL10 and CCL5 production in the peritoneal fluids of the infected mice (Figure 8). There was no significant difference in the production of CCL2 and CXCL10 in the peritoneal fluids of the infected WT and CCR5^−/−^ mice. However, significantly higher levels of CCL5 were observed in CCR5^−/−^ mice infected with RH-OE at 3 and 5 dpi, indicating CCL5 production took place in a TgCyp18-dependent and CCR5-independent way.

## Discussion

Control of acute toxoplasmosis relies on a potent Th1 cell response that requires IL-12 and IFN-γ production, which are generated through both innate and adaptive responses [21,22]. It appears that *Toxoplasma* is unique in that it possesses two mechanisms that trigger IL-12 production in DCs and macrophages [3,12,23]. One of these mechanisms is dependent upon the common adaptor protein MyD88, and is likely to involve TLR11 [3,10,23]. The other mechanism is dependent upon TgCyp18, which is released by extracellular tachyzoites, triggering IL-12 production through binding to CCR5 [12]. Recently, our group reported that TgCyp18 induced production of NO, TNF-α and IL-12p40 in macrophages, and also up-regulated the production of IFN-γ and IL-6 in these cells [13]. In the present study, the levels of IL-12 in the ascetic fluid of RH-OE-infected CCR5^−/−^ mice were significantly higher than those in RH-GFP-infected CCR5^−/−^ mice, suggesting that cytokine production arose via TgCyp18-dependent and CCR5-independent mechanisms. TgCyp18 stimulated IL-12 production in macrophages [13] and DCs [12]. Therefore, macrophages and DCs both play a role in IL-12 production in the present study. Further investigations are required to distinguish the relative contributions made by these cells. These results suggest that CCR5-independent accumulation of inflammatory cells at the site of infection might produce higher levels of pro-inflammatory cytokines in CCR5^−/−^ mice.

The ability of *T. gondii* to attract, invade, and survive inside immune cells (T cells, DCs and macrophages), along with the migratory properties of DCs and macrophages that allow parasite dissemination around the host have been reported previously [7,24]*[26]. Our results revealed that while *T. gondii* could infect CD3^+^, CD11c^+^, and CD11b^+^ cells, it exhibited a preference for CD11b^+^. We observed enhanced recruitment of CD11b^+^ cells after infection with RH-OE. This chemotactic effect of TgCyp18 was correlated with the ability of RH-OE to increase CCR5 expression levels. Thus, overproduction of TgCyp18 during RH-OE infection enhanced cellular recruitment. Recruitment of CD11b^+^ cells in CCR5^−/−^ mice infected with RH-OE was also higher than that in RH-GFP-infected mice. Additionally, there was no significant difference in the recruitment of CD11b^+^ cells between WT and CCR5^−/−^ mice that were infected peritoneally with RH-GFP tachyzoites. Recently, our group demonstrated that recombinant TgCyp18 controlled the *in vitro* migration of macrophages and lymphocytes in CCR5-dependent and -independent ways [14]. Therefore, the results presented here suggest that the TgCyp18-induced cell migration occurred in a CCR5-independent way in our *in vivo* experimental model.

Migration of macrophages and lymphocytes to the site of infection would enhance *T. gondii* invasion into these cells, after which the parasite-infected cells, such as CD11b^+^ leukocytes, are transported to other organs [7]. Our quantitative PCR analyses revealed that infection with RH-OE resulted in an increased parasitic load in the liver compared with RH-GFP infection. These results suggest that cells recruited by TgCyp18 are used to shuttle the parasite to other organs. In general, chemokines and their receptors play an important role in the migration of immune cells. A previous study showed that an early burst of CCR5 ligand production occurred in the tissue of WT and CCR5^−/−^ mice by day 5 after oral infection with *T. gondii* strain 76 k cysts [27]. Our present study showed that recombinant TgCyp18 increased the expression levels of CCL5 in macrophages. In addition, significantly higher levels of CCL5 were detected in the peritoneal fluids of CCR5^−/−^ mice infected RH-OE. Moreover, CCL5 levels in the livers of WT mice infected with RH-OE at 5 dpi were higher than those in the other experimental groups. Consistent with our findings, a previous study showed that the parasite numbers in the livers of CCR5^−/−^ mice were higher than those of the C57BL/6 wild-type animals, while the parasite numbers were similar in other organs of the WT and CCR5^−/−^ mice [27]. Therefore, TgCyp18-mediated CCL5 production might contribute to macrophage migration to the site of infection and the transport of *T. gondii*-infected cells to the liver. Besides CCR5, CCL5 has been shown to interact with other receptors, including CCR3 and CCR1. Therefore, activation of CCR1- and CCR3-signaling may contribute to CCL5-mediated pathology during *T. gondii* infection. Hence, the chemokines up-regulated in CCR5^−/−^ mice infected with RH-OE may play a crucial role in CCR5-independent macrophage migration.

To test this idea in our study, the expression levels of chemokines related to macrophage migration were investigated. *In vitro* analysis showed that TgCyp18 increased the expression of CCL6 in a CCR5 independent manner. However, the *in vivo* data showed that a higher level of CCL6 was observed in the livers of the CCR5^−/−^ mice infected RH-GFP at 3 dpi compared with those infected with RH-OE. Although we do not know the reason for the difference between the *in vitro* and *in vivo* data, it is possible that CCL6 expression might have been induced before 3 dpi in the livers of the CCR5^−/−^ mice infected with RH-OE. It is interesting to note that CCL2 expression was slightly increased in macrophages treated with recombinant TgCyp18. Moreover, the expression levels of CCL2 and CXCL10 were significantly higher at 3 dpi in the livers of CCR5^−/−^ mice infected with RH-OE compared with the uninfected mice. Thus, TgCyp18-mediated production of CCL2 and CXCL10 in the liver may trigger transport of *T. gondii*-infected macrophages via a CCR2 and CXCR3-dependent mechanism, respectively. CCR2^−/−^ mice have profound defects in monocyte recruitment although constitutive trafficking remains unaffected [28]. CCR2^−/−^ mice or CCL2^−/−^ mice failed to recruit Gr1^+^ inflammatory monocytes, which are required for mucosal resistance to *T. gondii*[29], or to control systemic toxoplasmosis by intraperitoneal infection [30]. Furthermore, another group reported that the CXCR3 ligands, CXCL9, CXCL10 and CXCL11, were induced markedly at the levels in the spleen, lung, and liver following infection with *T. gondii*[27]. Induction of these chemokines was similar in WT and CCR5^−/−^ mice up to day 5 [27]. CXCL10 is required to maintain T-cell populations and to control parasite replication during chronic ocular toxoplasmosis [31]. These results suggest that CCR2 and CCL2, or CXCR3 and its ligands, play a crucial role in cell migration and control of *T. gondii* infection.

Diana et al. [32] showed that a *T. gondii* excreted-secreted antigen induced recruitment and migration of human DCs in a CCR5-dependent fashion. Other studies in mice have reported that *T. gondii* activates DCs and triggers their migration to the spleen to activate proliferation of T cells, or to potentiate parasite dissemination [25,33]. TgCyp18 can attract mouse DCs *in vitro*[12]. CCR5 plays an important role in the migration of intraepithelial CD8^+^ T cells, and in the regulation of an inflammatory response following *T. gondii* infection [8]. CCR5 also has a role in the migration of NK cells, with severe deleterious effects observed in infected mice [27]. Thus, it has been shown that increased immune cell migration is involved in the pathogenesis and control of infection with *T. gondii*. In the present study, based on survival rates, significant differences were not detected in the parasite-challenged (RH-WT, RH-GFP and RH-OE) mice (data not shown). All mice (*n* = 6) infected intraperitoneally with 1,000 tachyzoites died by 8–9 dpi. All mice (*n* = 4) infected intraperitoneally with 100 tachyzoites died by 11–15 dpi. Histopathological lesions in livers, spleens and lungs were observed in all mice infected with RH-GFP and RH-OE, but there were no remarkable differences in the severity of the lesions among the experimental groups (Additional file 2: Figure S2). This was probably related to the high virulence of the *T. gondii* type I strain. In addition, to determine whether macrophages assisted with *T. gondii* dissemination in the mice, C57BL/6 mice were subject to macrophage depletion by treatment with clodronate liposome, and then challenged with the *T. gondii* PLK strain (type II). The survival rates of the clodronate-treated and untreated mice were 71% and 43% (n = 7), respectively. Therefore, it appears likely that macrophages assisted with *T. gondii* dissemination in the mice*.* However, the pathogenesis of infection with the RH strain is quite different from that of infection with the PLK strain. Hence, further investigations are required to confirm the contribution of TgCyp18 to parasite pathogenesis and the role of macrophages in parasite dissemination.

The recombinant strain (RH-OE) of the parasite expresses TgCyp18 fused to HA. Therefore, it is unclear whether the effects of infection with RH-OE were due to TgCyp18 or HA (or both). To address this, we generated a recombinant *T. gondii* parasite that expressed the TgCyp18-HA fusion protein as mutants (^17^GEH^19^ to ^17^AAA^19^ and ^149^RP^150^ to ^149^YV^150^), which when tested, exhibited reduced interactions with CCR5 (RH-DN, Additional file 3: Figure S3). There was no significant difference in IL-12 production levels in ascites fluid and recruitment of immune cells between the mice infected with RH-GFP and RH-DN (Additional file 4: Figure S4). Therefore, these data suggest that the effects of infection with RH-OE were not due to the HA tag. In addition, the interaction between TgCyp18 and CCR5 played a role in IL-12 production and recruitment of immune cells in the wild type mice.

Taken together, it appears that TgCyp18 might enhance its effects directly through binding with CCR5 and/or another receptor or receptors not yet identified. Regarding TgCyp18-CCR5 dependent or independent responses, there are two explanations that might clarify our observations. First, optimal production of TgCyp18 may under normal circumstances work on CCR5 and/or other receptor(s) to recruit immune cells that produce cytokines. This possibility seems obvious in view of our previous results that showed that TgCyp18 controlled the *in vitro* migration of macrophages and spleen cells in a CCR5-dependent manner [14]. In contrast, TgCyp18 may initiate cytokine production and macrophage proliferation in a CCR5-independent manner [13,14]. Second, it is possible that stimulation of host cells with TgCyp18 via CCR5 and/or other receptor(s) could trigger expression of chemokine receptors and its ligands for cell migration. Increased CCL5 levels in the livers of the wild-type mice infected with RH-OE parasites indicates that parasite migration to this organ occurred in a TgCyp18- and CCR5-dependent manner. Furthermore, parasite migration, which occurred in a CCR5-independent and TgCyp18-dependent way, can be explained by the higher levels of CCL2 and CXCL10 in the liver and CCL5 in the ascites fluid of CCR5^−/−^ mice infected with RH-OE. Thus, the present results suggest that TgCyp18 has the ability to enhance host-cell migration via CCL5 and parasite dissemination by CCL2 and CXCL10 in a CCR5-independent manner.

## Conclusion

We determined that TgCyp18 plays a crucial role in the migration of CD11b^+^ cells to the site of *T. gondii* infection, and that the mechanisms responsible could be both dependent on and independent of CCR5 expression levels. Enhanced migration of host cells will mediate *T. gondii* transport to organs, especially the liver. We have shown that there are several options available to *T. gondii* for completing its infection cycle, one of which is CCR5-dependent, others of which involve TgCyp18-mediated production of chemokines in a CCR5-independent manner. Additional work will be required to clarify the precise role that TgCyp18 plays in parasite-infected host cells and in parasite migration in the host.

## Competing interests

The authors declare that they have no competing interests.

## Authors’ contributions

YN and HMI designed the study and prepared this manuscript. HMI, MN, ST, WA and YN performed the experiments. HMI, HF, XX and YN analyzed the results. All authors have read and approved the final manuscript.

## Supplementary Material

Additional file 1: Figure S1 Absolute number of immune cells in the ascites fluid of mice. WT and CCR5^-/-^ (KO) mice were infected intraperitoneally with *T. gondii* tachyzoites. At 5 dpi, peritoneal cells were subjected to flow cytometry to determine the number of GFP^+^ host cells. Each value represents the mean ± the standard deviation of four replicate samples. Click here for file

Additional file 2: Figure S2 Histopathological lesions in mouse tissues infected with *T. gondii* RH-OE and RH-GFP at 5 days after infection. Tissues were fixed in 10% formalin solution. After fixation, they were embedded in paraffin wax, sectioned to 4 μm, and then stained with hematoxylin and eosin (HE). (A, B) Liver, focal inflammatory cell infiltration was found in all groups. (C, D) Spleen, mononuclear cell infiltration in serosa and fat tissue (arrow-head in C and detail in inset). (E, F) Lung, slight to mild inflammatory cell infiltration. Histopathological findings were similar in both groups. Multifocal inflammatory cell infiltration was found in the liver. In the spleen, no significant changes were observed in parenchyma, however mononuclear cell infiltration was observed in serosa and fat tissue, which indicated peritonitis. Also, slight to mild inflammatory cell infiltration was found in the lung tissue. Click here for file

Additional file 3: Figure S3 TgCyp18 mutants, namely ^17^GEH^19^ to ^17^AAA^19^ and ^149^RP^150^ to ^149^YV^150^, which are located in the N and C termini of the protein, respectively, had reduced interactions with CCR5 [15]. To generate TgCyp18 mutants, primers containing an *Eco*RV site (boldface) (5'-CAT G**GA TAT C**GA CAT CGA CGC AGC AGC TGC-3') and a *Nru*I restriction site (boldface) (5'-CCG TGA TTT **TCG CGA** CCT TAG ACA CGT AGC-3') were used. Amplicons were digested with *Eco*RV and *Nru*I and then ligated into pCR4-TOPO-TgCyp18, which had been treated with *Eco*RV and *Nru*I to give pCR4-TOPO-MTgCyp18. pCR4-TOPO-MTgCyp18 was digested with *Nco*I and *Nhe*I and the resulting products ligated into pHXNTPHA, resulting in the plasmid, pHXNTP-MTgCyp18HA. The coding sequence corresponding to the full-length TgCyp18 mutant fused to HA (MTgCyp18-HA) was obtained from pHXNTP-MTgCyp18HA by *Nco*I and *Bgl*II digestion. Liberated fragments were treated with the Klenow fragment and inserted into the *Eco*RV site of pDMG. The pDMG-MTgCyp18HA vector contained expression cassettes for GFP, DHFR-TS and MTgCyp18-HA. The resultant recombinant *T. gondii* clones of pDMG-MTgCyp18HA were designated RH-DN. Western blot analysis of *T. gondii* tachyzoite of RH-DN clones (C1, C2, C3) including RH-WT and RH-OE clones (C1, C2 and C3) was performed. Because the RH-DN C3 clone expressed high levels of MTgCyp18-HA it was selected for further study. Click here for file

Additional file 4: Figure S4. (A) IL-12 production in the ascites fluid of infected mice. Wild type mice were infected intraperitoneally with *T. gondii* tachyzoites. At 3 and 5 days post-infection (dpi), IL-12 production in the ascites fluid was measured. Each value represents the mean ± the standard deviation of four replicate samples. A significant increase in IL-12 production was seen in the mice infected with RH-OE at 3 dpi compared with those infected with RH-GFP or RH-DN. (B) Recruitment of immune cells. Wild type mice were infected intraperitoneally with *T. gondii* tachyzoites. At 3 days post-infection (dpi), peritoneal cells were harvested from uninfected or parasite-infected mice. Cells were then subjected to flow cytometry to determine the absolute number of cells expressing CCR5, CD11b, CD11c, or CD3. Each value represents the mean ± the standard deviation of four replicate samples. RH-OE infection enhanced the recruitment of CD11b^+^, CCR5^+^, and CD3^+^ cells compared with RH-GFP or RH-DN infections. Click here for file
