# Supplementary figures and images for: Overproduction of Toxoplasma gondii cyclophilin-18 regulates host cell migration and enhances parasite dissemination in a CCR5-independent manner
Source: BMC Microbiol. 2014 Mar 25;14:76. doi: 10.1186/1471-2180-14-76 (PMC3987834; doi:10.1186/1471-2180-14-76)

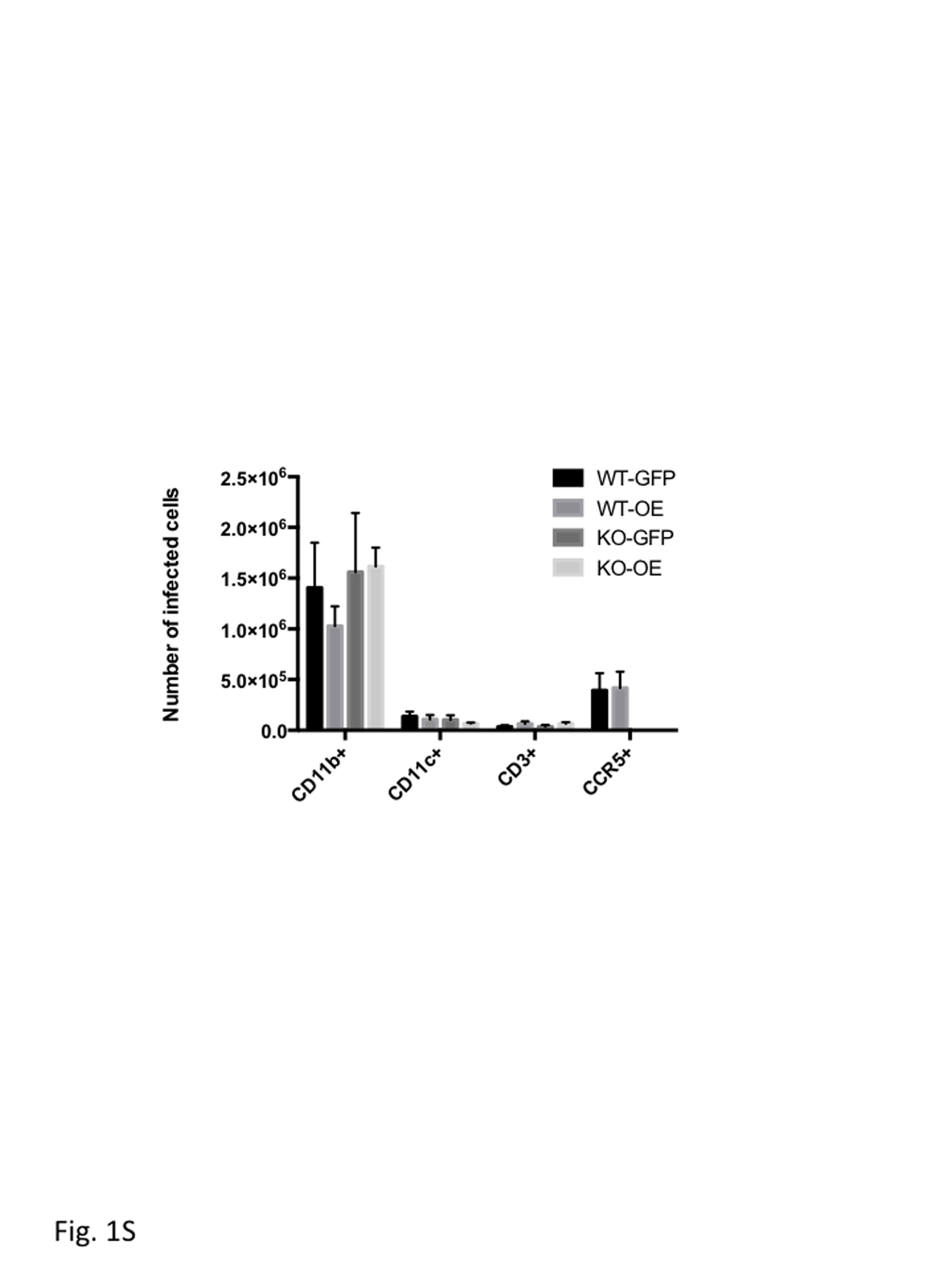

Supplement: Additional file 1: Figure S1 — Absolute number of immune cells in the ascites fluid of mice. WT and CCR5-/- (KO) mice were infected intraperitoneally with T. gondii tachyzoites. At 5 dpi, peritoneal cells were subjected to flow cytometry to determine the number of GFP+ host cells. Each value represents the mean ± the standard deviation of four replicate samples. [file 1471-2180-14-76-S1.tiff]

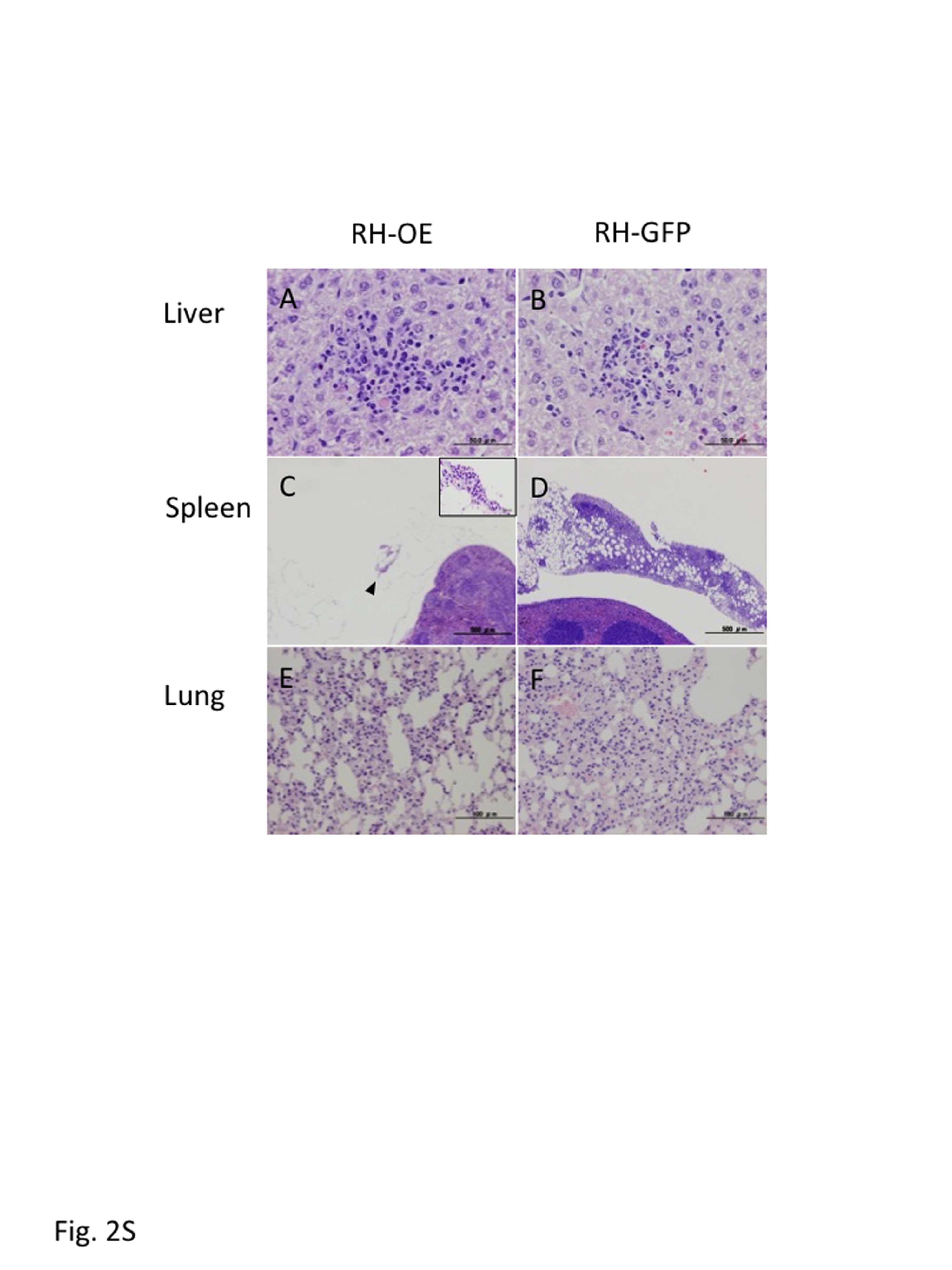

Supplement: Additional file 2: Figure S2 — Histopathological lesions in mouse tissues infected with T. gondii RH-OE and RH-GFP at 5 days after infection. Tissues were fixed in 10% formalin solution. After fixation, they were embedded in paraffin wax, sectioned to 4 μm, and then stained with hematoxylin and eosin (HE). (A, B) Liver, focal inflammatory cell infiltration was found in all groups. (C, D) Spleen, mononuclear cell infiltration in serosa and fat tissue (arrow-head in C and detail in inset). (E, F) Lung, slight to mild inflammatory cell infiltration. Histopathological findings were similar in both groups. Multifocal inflammatory cell infiltration was found in the liver. In the spleen, no significant changes were observed in parenchyma, however mononuclear cell infiltration was observed in serosa and fat tissue, which indicated peritonitis. Also, slight to mild inflammatory cell infiltration was found in the lung tissue. [file 1471-2180-14-76-S2.tiff]

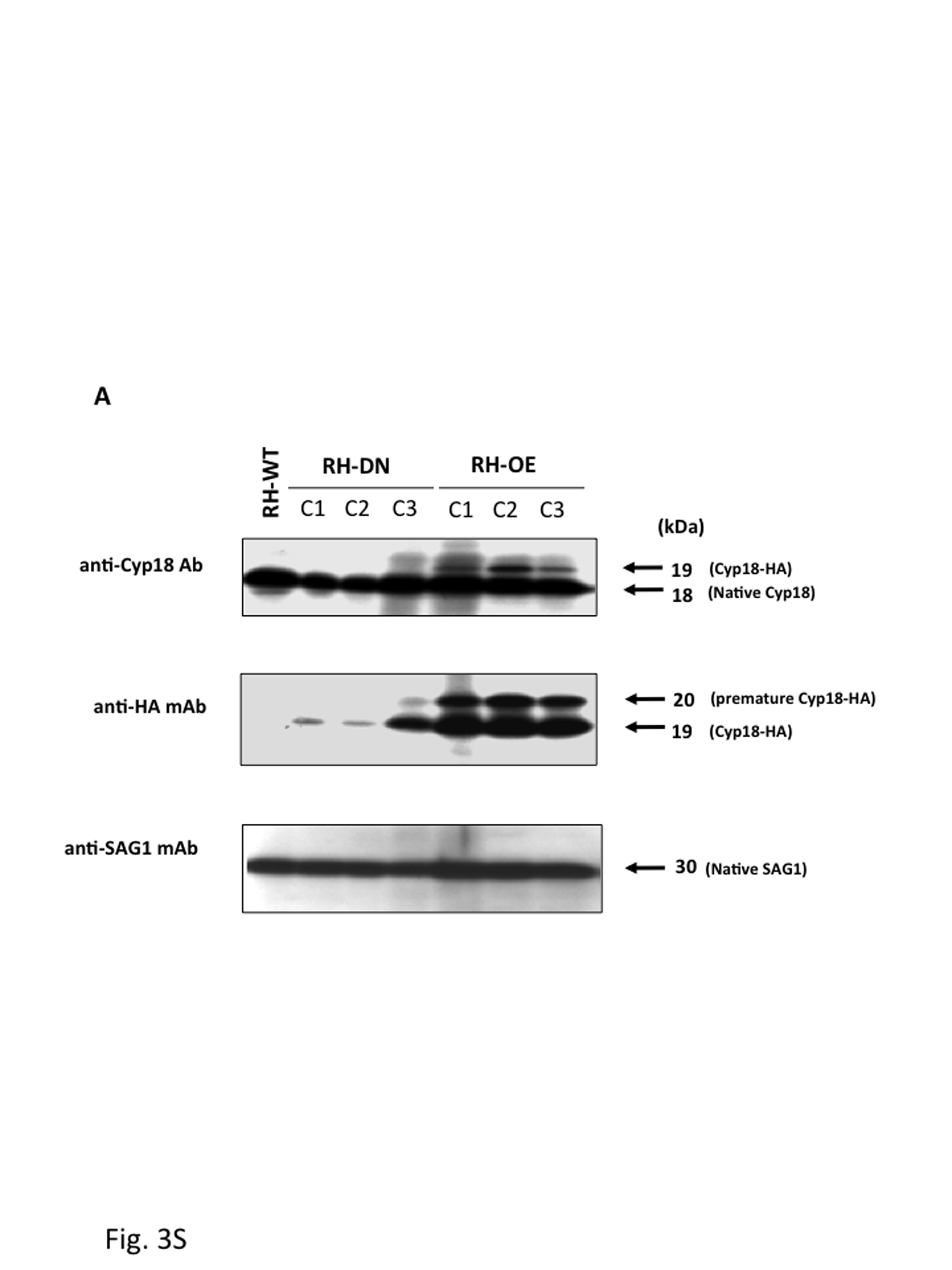

Supplement: Additional file 3: Figure S3 — TgCyp18 mutants, namely 17GEH19 to 17AAA19 and 149RP150 to 149YV150, which are located in the N and C termini of the protein, respectively, had reduced interactions with CCR5 [15]. To generate TgCyp18 mutants, primers containing an EcoRV site (boldface) (5'-CAT GGA TAT CGA CAT CGA CGC AGC AGC TGC-3') and a NruI restriction site (boldface) (5'-CCG TGA TTT TCG CGA CCT TAG ACA CGT AGC-3') were used. Amplicons were digested with EcoRV and NruI and then ligated into pCR4-TOPO-TgCyp18, which had been treated with EcoRV and NruI to give pCR4-TOPO-MTgCyp18. pCR4-TOPO-MTgCyp18 was digested with NcoI and NheI and the resulting products ligated into pHXNTPHA, resulting in the plasmid, pHXNTP-MTgCyp18HA. The coding sequence corresponding to the full-length TgCyp18 mutant fused to HA (MTgCyp18-HA) was obtained from pHXNTP-MTgCyp18HA by NcoI and BglII digestion. Liberated fragments were treated with the Klenow fragment and inserted into the EcoRV site of pDMG. The pDMG-MTgCyp18HA vector contained expression cassettes for GFP, DHFR-TS and MTgCyp18-HA. The resultant recombinant T. gondii clones of pDMG-MTgCyp18HA were designated RH-DN. Western blot analysis of T. gondii tachyzoite of RH-DN clones (C1, C2, C3) including RH-WT and RH-OE clones (C1, C2 and C3) was performed. Because the RH-DN C3 clone expressed high levels of MTgCyp18-HA it was selected for further study. [file 1471-2180-14-76-S3.tiff]

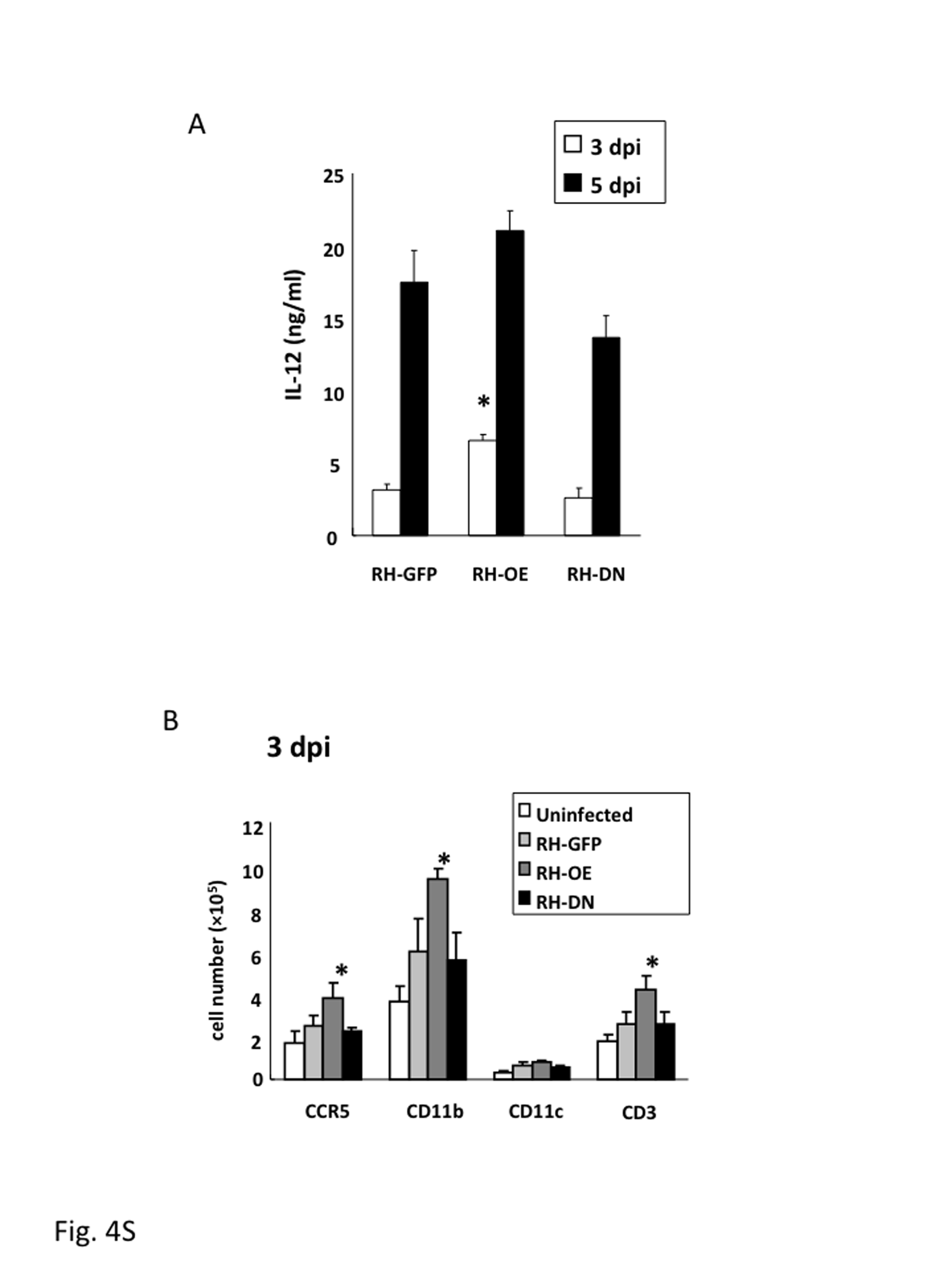

Supplement: Additional file 4: Figure S4. — (A) IL-12 production in the ascites fluid of infected mice. Wild type mice were infected intraperitoneally with T. gondii tachyzoites. At 3 and 5 days post-infection (dpi), IL-12 production in the ascites fluid was measured. Each value represents the mean ± the standard deviation of four replicate samples. A significant increase in IL-12 production was seen in the mice infected with RH-OE at 3 dpi compared with those infected with RH-GFP or RH-DN. (B) Recruitment of immune cells. Wild type mice were infected intraperitoneally with T. gondii tachyzoites. At 3 days post-infection (dpi), peritoneal cells were harvested from uninfected or parasite-infected mice. Cells were then subjected to flow cytometry to determine the absolute number of cells expressing CCR5, CD11b, CD11c, or CD3. Each value represents the mean ± the standard deviation of four replicate samples. RH-OE infection enhanced the recruitment of CD11b+, CCR5+, and CD3+ cells compared with RH-GFP or RH-DN infections. [file 1471-2180-14-76-S4.tiff]
